# MACHINE LEARNING MODELING OF LOW QUALITY SURVEY RESPONSES TO PREDICT COGNITIVE IMPAIRMENT AMONG OLDER ADULTS

**DOI:** 10.1093/geroni/igae098.1011

**Published:** 2024-12-31

**Authors:** Hongxin Gao, Stefan Schneider, Raymond Hernandez, Jenny Harris, Danny Maupin, Doerte U Junghaenel, Haomiao Jin

**Affiliations:** University of Surrey, Guildford, England, United Kingdom; University of Southern California, Los Angeles, California, United States; University of Southern California, Los Angeles, California, United States; University of Surrey, Guildford, England, United Kingdom; University of Surrey, Guildford, England, United Kingdom; University of Southern California, Los Angeles, California, United States; University of Surrey, Guildford, England, United Kingdom

## Abstract

Early identification of cognitive impairment (CI) is critical for managing Alzheimer’s disease and other dementia. Leveraging emerging evidence on the relationship between subtle errors in survey responses and CI, this study uses a novel informatics approach that combines machine learning with psychometric methods to develop a risk prediction model for identification of CI, including mild CI and dementia, in the general older adult population. The study is based on a sample of 12,942 participants aged 50 and above in the Health and Retirement Study, and psychometric indices of low-quality responses (LQR) in a range of different surveys are created as predictors. Our analysis shows an area under the curve (AUC) of 0.66 for identifying current CI and 0.70 for predicting dementia or mortality in the next 10 years. Also, the subgroup analysis shows the LQR indices have better predictive performance in the 50-59 (AUC=0.72), and 60-69 (AUC=0.71) age groups, suggesting the model may be more sensitive to early cognitive deficits. A unique feature of this tool is that it does not require the underlying surveys to be directly relevant to CI; thus, health professionals, especially those working in community settings like health and social workers, may use the tool to assist identifying older adults at risk of CI based on questionnaires of other aspects of their life, such as quality of life and personality. It may also be useful for aging researchers who intend to identify high-risk populations from survey data that do not include direct assessment of CI.

